# Structural and Chemical Dynamics of Cu/NiO/Cu(100) Surfaces During CO_2_ Hydrogenation

**DOI:** 10.1002/anie.8933772

**Published:** 2026-09-08

**Authors:** Mauricio J. Prieto, Hao Wan, Liviu C. Tănase, Lucas de Souza Caldas, Aarti Tiwari, Karsten Reuter, Hendrik H. Heenen, Vanessa J. Bukas, Thomas Schmidt, Beatriz Roldan Cuenya

**Affiliations:** ^1^ Department of Interface Science Fritz‐Haber‐Institut Der Max‐Planck‐Gesellschaft Berlin Germany; ^2^ Theory Department Fritz‐Haber‐Institut der Max‐Planck‐Gesellschaft Berlin Germany

**Keywords:** chemical state, CO_2_ hydrogenation, CuNi, LEEM, NAP LEEM, NAP PEEM, PEEM, segregation

## Abstract

Understanding the dynamic restructuring of bimetallic catalysts under reaction conditions is key to improving their performance and stability. Here, we unveil the drastic changes in the structure and surface composition of copper‐encapsulated NiO islands supported on Cu(**100**) during CO_2_ hydrogenation. Using in situ low‐energy electron microscopy (LEEM), x‐ray photoemission electron microscopy (XPEEM), and spatially resolved x‐ray absorption spectroscopy (µ‐NEXAFS), we show that NiO gradually reduces under hydrogenation conditions, despite being buried beneath a ∼3 nm Cu overlayer. Furthermore, the reduced Ni exhibits distinct spatial redistribution depending on whether CO is present in the CO_2_/H_2_ reaction mixture. Density functional theory (DFT) calculations establish an adsorbate‐dependent rule: C‐bound intermediates thermodynamically favor Ni segregation, whereas O‐bound species stabilize a Cu‐terminated surface. Near‐ambient pressure (NAP) LEEM directly visualizes size‐dependent NiO island reduction governed by adsorbed H_ads_ availability and local geometry. Together, these results show how reaction microenvironments can be used to steer bimetallic surface composition and the active sites during CO_2_ hydrogenation.

## Graphical abstract

Dynamic segregation as a design principle for Cu–Ni catalysts. Operando LEEM/XPEEM tracks reduction, reshaping, and surface segregation processes taking place in Cu‐encapsulated NiO islands supported on Cu(**100**) during CO_2_ hydrogenation.

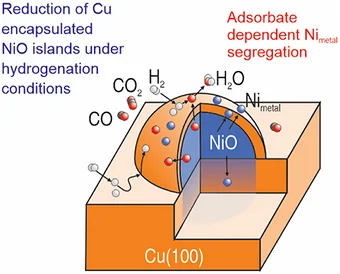

## 1 Introduction

Catalyst surfaces are not static under reaction conditions [1, 2, 3, 4]. Their composition, oxidation state, and morphology can adapt on the fly, reshaping the identity of the active sites. For bimetallic catalysts, this creates a central challenge when linking mass transport and segregation processes under reaction conditions to the chemistry of surface intermediates. Establishing mechanistic descriptors that predict when a reactive component emerges at the surface or dissolves below it is critical for designing catalysts that remain selective under changing feeds.

Carbon dioxide capture and reutilization constitute fundamental strategies to mitigate the adverse climate effects of current emissions. In CO_2_ hydrogenation, capturing the former mentioned dynamics requires operando, surface‐sensitive methods capable of tracking the surface composition and morphology in real time. In combination with green hydrogen, CO_2_ hydrogenation can provide key platform chemicals of high industrial value while closing the carbon cycle. Among these, methanol remains an extremely attractive product when considering applications in industry. Because of its versatility, methanol remains an attractive building block for derived products, including ethylene and propylene [5, 6].

Industrial methanol production from syngas over Cu/ZnO/Al_2_O_3_ is well‐established [7, 8], but recent efforts intensified the search for catalysts that remain active, selective, and stable under more flexible reaction conditions. Bimetallic systems such as CuNi have therefore attracted attention due to their enhanced performance compared to monometallic counterparts, often attributed to alloying‐related synergistic effects [9]. However, the mechanistic role of each component under dynamic reaction environments remains incompletely understood [10, 11].

Mechanistically, while methanol formation from CO has been reported over Cu/ZnO [12] and Ni/Cu(hkl) surfaces [13], it is widely accepted that CO_2_ is the dominant carbon source in syngas feeds [14, 15]. The reaction network is rather complex [16, 17, 18], leading to a broad optimization space in activity, stability, and selectivity. Thus, catalyst performance can be impacted by its morphological evolution [19], site blocking [20, 21], recrystallization/sintering and changes in Cu oxidation state [22, 23, 24], among other factors [25].

A striking example of the interplay between adsorbates and surface composition is the reported ∼ 60 times increase in methanol yield only when Ni and CO are concomitantly present in Cu‐based catalysts [13, 26, 27], indicating that CO can influence both the reaction network and the operative surface state. CO is a key intermediate and, depending on conditions, its slow hydrogenation can render C─O bond scission resulting in poisoning of the active sites; co‐dosing CO can shift equilibria and suppress this detrimental path [28, 29, 30, 31]. In addition, CO may act as an oxygen scavenger via the water‐gas‐shift parallel reaction, affecting the steady‐state abundance of oxygenated surface species [32]. Because O and CO_2_ can compete for metallic Cu sites, removing oxygenated species can facilitate the regeneration of the active sites [14, 29]. Water adds another layer of complexity: while H_2_O can inhibit the reaction at high conversions, small amounts may stabilize promoted Cu–ZnO_1__−__x_ by preventing over‐reduction [21, 33].

Beyond kinetics, the dynamic adaptation of the surface composition in multimetallic catalysts is central to understanding CuNi performance. The role of CO has often been discussed as stabilizing Ni in the topmost layer of the catalyst, yet near ambient pressure (NAP) studies on size‐selected NiCu nanoparticles (NPs) [10, 34] revealed that the surface composition depends strongly on the gaseous environment. In particular, Cu surface enrichment was detected under CO_2_/CO/H_2_ compared to CO_2_/H_2_, accompanied by increased methanol selectivity—a counter intuitive outcome if only the CO binding strength is considered. DFT‐based segregation‐energy analyses have linked this behavior to the combined adsorption of reactants and intermediates, but the underlying mass‐transport pathways under reaction conditions remain unclear. Moreover, in prior studies, all spectroscopy data presented corresponded to ensemble‐averaged spectroscopic methods, which obscures the various processes that might be occurring on an intrinsically heterogeneous catalyst surface.

Here, we address this knowledge gap by combining locally resolved spectroscopy and microscopy with DFT to determine how reaction microenvironments steer the surface composition of catalysts. We monitor the evolution of the structure and surface composition of Cu/NiO/Cu(**100**) catalysts under CO_2_ hydrogenation conditions using in situ low‐energy electron microscopy (LEEM), x‐ray photoemission electron microscopy (XPEEM), and µ‐NEXAFS. Complementary DFT calculations quantify adsorbate‐driven energetics and connect intermediate binding motifs to surface termination. Our results provide operando evidence for a feed‐dependent switch in Ni segregation.

## 2 Results and Discussion

### 2.1 Preparation of the Cu/NiO/Cu(**100**) System

Figure 1 summarizes the structural and chemical state of the Cu/NiO/Cu(**100**) system. The pristine Cu(**100**) surface (Figure 1a) shows well‐defined terraces with step edges and bunches, and a sharp LEED pattern (inset), confirming the surface order. Ni deposition at room temperature onto the clean Cu substrate does not result in observable 3D island formation (see Figure S1), consistent with pseudomorph growth reported in the literature [35]. A rather weak c(2×2) phase is observed, probably derived from the adsorption of background gases like CO and O_2_ [36, 37].

**FIGURE 1:**
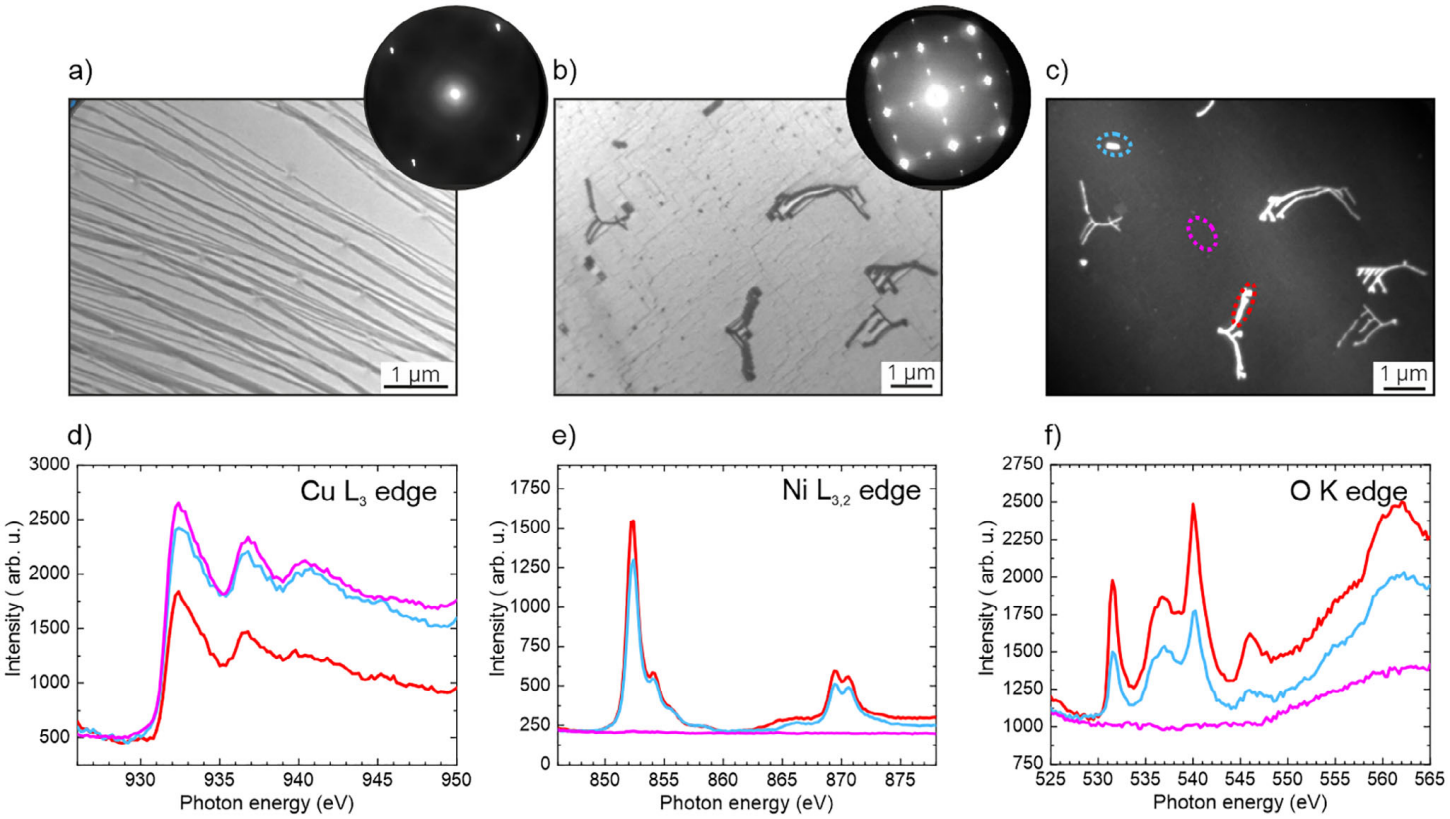
(a) LEEM images of pristine Cu(**100**) collected with an electron energy (E_0_) of 5 eV. Inset: µLEED pattern collected with *E*_0_ = 42 eV. (b) LEEM image showing NiO islands on Cu(**100**) obtained by annealing at 820 K in 5×10‐6 mbar O_2_ for 10 min. Inset: µLEED pattern collected from the area displayed in the LEEM image in (b). (c) XPEEM image of the sample in (b) collected at using secondary electrons at a photon energy of 852 eV. (d–f) Local NEXAFS spectra revealing the elemental distribution of Cu, Ni, and O across the sample surface collected on different regions color‐coded in (c).

Oxidation and Ni segregation were triggered by annealing the Ni/Cu(**100**) surface in 5×10^−6^ mbar O_2_ at 820 K for 10 min. The preferential oxidation and segregation of Ni during this treatment is consistent with the stronger oxophilicity of Ni relative to Cu. In the framework proposed by Campbell and coworkers [38], stronger oxophilicity reflects stronger metal–oxygen interfacial bonding and therefore greater thermodynamic stabilization of oxidized Ni species under oxygen‐rich environments. The LEEM image in Figure 1b reveals significant step rearrangement and a rectangular surface reconstruction that persists upon sample cooling. NiO islands preferentially form along these reconstructions, indicating a correlation between the substrate morphology and island growth.

It is important to note that various island morphologies (cubic, triangular, and elongated) are observed in our experiments under identical experimental conditions, illustrating the intrinsic heterogeneity of the system before CO_2_ hydrogenation, as it is typically observed in industrial materials. Figure S2 shows LEEM images collected for different samples to illustrate this point. Step edges/bunches on the Cu substrate act as diffusion barrier for Ni species, limiting island growth more by Ni mobility than by O_ads_ availability. Figure S1 shows extensive Cu step movement at 820 K, near the reported surface melting temperature for the (100) facet (∼ 870‐900 K) in line with high atomic mobility [39, 40]. Interestingly, once formed, NiO islands pin step edges, consistent with the lower mobility of cationic Ni species and the high stability of the oxide phase. LEEM and µ‐LEED data reveal that the NiO islands grow preferentially laterally elongated in the [110] crystallographic direction.

X‐ray photoemission microscopy (XPEEM) local near edge x ray absorption spectra (µ‐NEXAFS) analysis (Figure 1c–f) confirm that Ni is localized within the islands and is absent between them. The Ni L_2,3_ edge shape matches that of high spin Ni^2+^ in bulk NiO. The O K‐edge also exhibits the characteristic NiO transitions (530–633 eV), along with the higher‐energy features consistent with ordered NiO nanostructures [41, 42, 43, 44]. The Cu L_3_ edge (Figure 1d) reveals invariancy across the sample. Comparing these spectra with known references for Cu, Cu_2_O, and CuO species [45, 46], allow us to infer the presence of Cu(I) species. More specifically, linear combination analysis (LCA) of the spectra reveals an average amount of 3% of Cu(I) species at this stage, in agreement with the incipient formation of Cu_2_O also indicated by the $\left(2\sqrt{2}\times \sqrt{2}\right)R45^{\circ }$ LEED pattern observed (inset) [47, 48, 49].

X‐ray photoelectron emission spectra (XPS) (Figure 2) show no Ni 2p signal in the pristine state, consistent with the encapsulation of the NiO islands with a Cu shell. By comparing the probing depths of both techniques (NEXAFS: 10 nm vs. XPS: 1.5 nm), we inferred a minimum Cu shell thickness of 3 nm. Prior studies of the adsorption of O_2_ on copper‐covered Ni(100) surfaces [50] indicated that oxygen can be readily adsorbed on the nickel surface. Exposure to large amounts of O_2_ can lead to the permeation of oxygen through the Cu overlayer. In our case, the large oxygen exposures employed (4000–5000 L range vs. 100 L in Ref. [50]) lead to a favorable condition for permeation, thus allowing the formation of NiO islands beneath the Cu active layer and highlighting the dynamic nature of the process of island formation.

**FIGURE 2:**
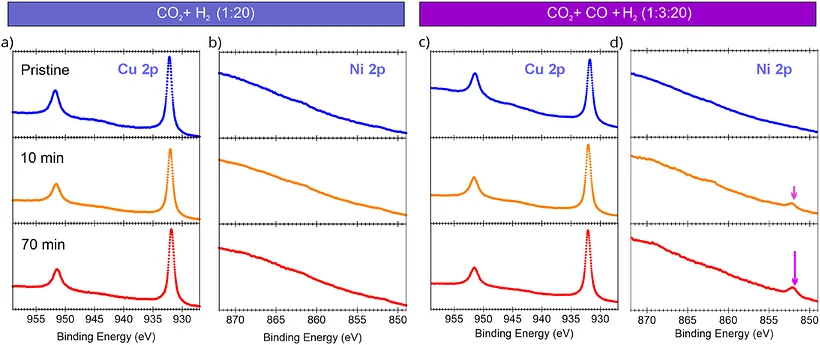
Photoemission spectra of Cu 2p (a, c) and Ni 2p (b, d) core levels collected after different sample treatments with photon energies of 1030 and 940 eV, respectively. Reaction times indicated in insets. An inelastic mean free path of 0.5 nm for both elements is estimated under the employed experimental conditions.

Table S1 in Supporting Information shows the positions of the 2p_3/2_ photoemission lines for Cu (932.0 ± 0.3 eV) and Ni (851.9 ± 0.3 eV) (when detectable). No significant variation is observed in the position of these lines across the treatments, regardless of the presence of CO in the environment. The Cu 2p_3/2_ binding energy is in agreement with the presence of Cu and/or Cu(I) species [51], which we cannot distinguish solely based on these data.

The sample state described so far constitutes the initial stage of our CO_2_ hydrogenation experiments. Scheme 1 summarizes the reaction conditions to which the NiO/Cu(**100**) samples were subjected before and during CO_2_ hydrogenation. Samples were submitted to accumulative reaction times in different CO_2_/H_2_ atmospheres in the absence and in the presence of CO.

**SCHEME 1:**
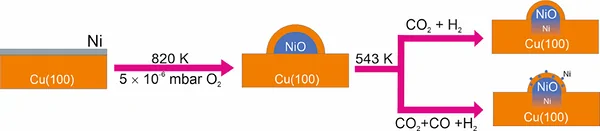
Model depicting the preparation of the NiO/Cu(**100**) model system used as starting catalyst surface in the CO_2_ hydrogenation experiments.

### 2.2 CO_2_ Hydrogenation on Cu/NiO/Cu(**100**): CO Effect

The reduction of NiO under the Cu skin proceeds regardless of the presence of CO in the gas phase, but CO controls whether the reduced Ni becomes detectable within the Cu encapsulating shell. Figure 3 presents a series of LEEM snapshots taken at different stages of the Cu/NiO/Cu(**100**) sample exposure to CO_2_ hydrogenation under varied gas composition. Two main features are evident in the temporal evolution of the surface morphology. First, the $\left(2\sqrt{2}\times \sqrt{2}\right)R45^{\circ }$ reconstruction of the Cu(**100**) surface is lifted upon exposure to the reaction mixture, with and without CO (see LEED patterns in Figure S3). LEEM further reveals the increasing curviness in the atomic steps with extended reaction time. This sluggish reconstruction lifting is attributed to the relatively low reaction temperature and the resulting low step mobility. Interestingly, after 10 min of reaction, the LEED pattern exhibits only the Cu(**100**) signature, while LEEM indicates that the NiO island morphology remains unchanged.

**FIGURE 3:**
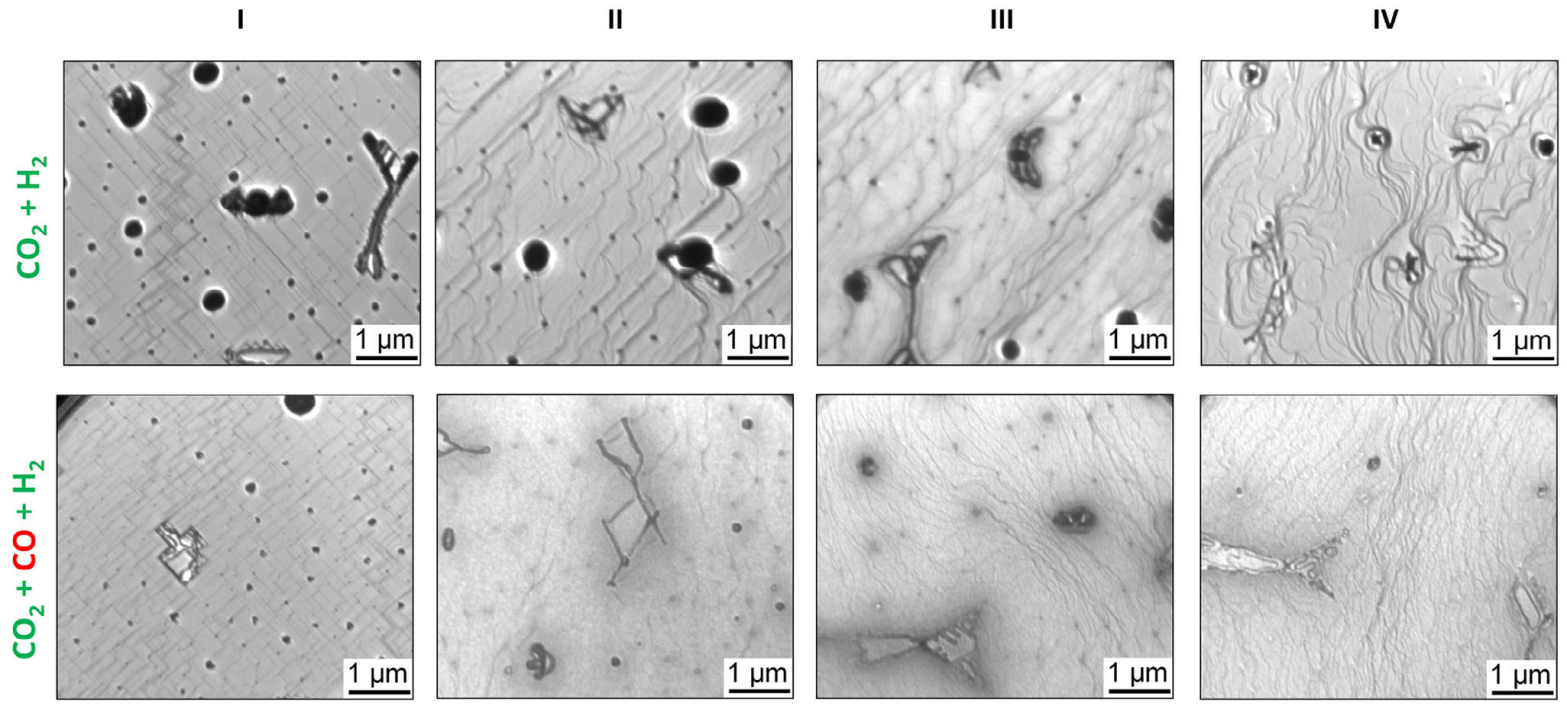
LEEM snapshots taken with *E*_a_ = 4 eV at different stages of the Cu/NiO/Cu(**100**) sample exposure to CO_2_ hydrogenation conditions. I, II, III, and IV correspond to accumulative reaction times of 0, 10, 30, and 75 min. Top panel: (1:20) CO_2_ + H_2_ reaction mixture (*p*_T_ = 2.1 mbar). Bottom panel: (1:3:20) CO_2_ + CO + H_2_ mixture (*p*_T_ = 2.4 mbar). Reaction was executed at 543 K.

A clear trend in the LEED patterns is evident with cumulative exposure (see Figure S3). In the absence of CO, only the lifting of the reconstruction is observed. However, the introduction of CO leads to the emergence of additional diffraction spots whose intensity increases with time. While the origin of these structures is not easily determined, two plausible explanations are proposed: (i) the formation of an ordered CO adlayer on Cu(**100**), or (ii) a restructuring of the Cu skin layer encapsulating the NiO islands. Prior studies under UHV conditions (10^−7^ mbar) suggests that scenario (i) is unlikely at room temperature [52]. In contrast, scenario (ii) implies significant mass transport of Cu even at moderate temperatures. Indeed, roughening of Cu(**100**) under CO exposure has been reported under NAP conditions, consistent with Cu atoms expulsion from the surface and the formation of small clusters [52]. In the present case, the broad LEED spot size suggest that such Cu domains are small (ca. 2 nm). Regardless of the origin of these spots, their emergence only in CO‐containing feeds indicates CO‐induced restructuring of the Cu skin, consistent with the subsequent appearance of a Ni signal in XPS only when CO is present (Figure 2).

Second, regardless of the reaction composition, encapsulated NiO islands shrink progressively during the reaction, with no significant difference in the reduction rate between the CO‐containing and CO‐free environments. Figure 4 presents a comparison of the NEXAFS data tracking the evolution of the local Cu and Ni electronic states for the pristine and 70 min reacted samples.

**FIGURE 4:**
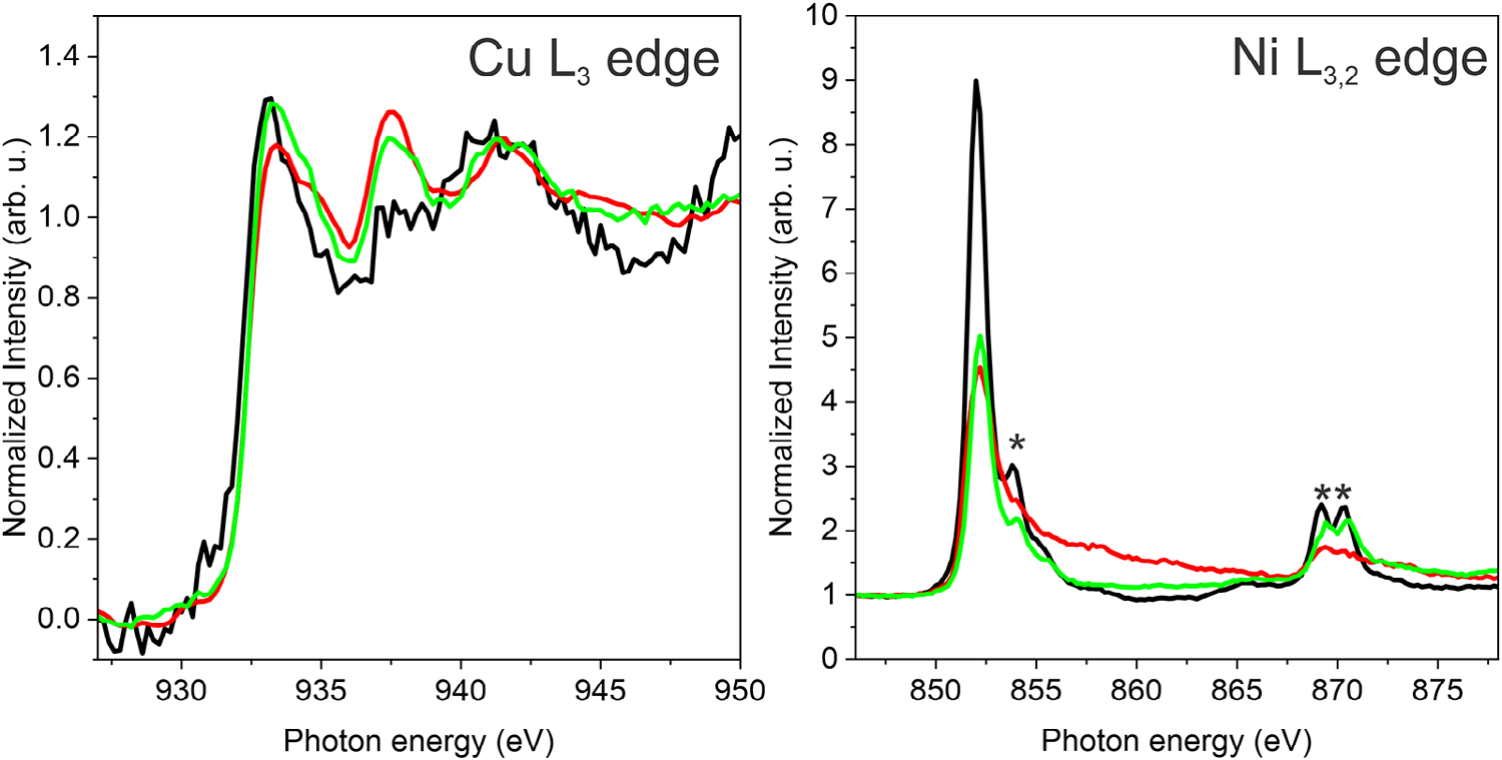
Local Cu and Ni L edge NEXAFS spectra collected at different stages of CO_2_ hydrogenation at 543 K. Spectra collected after subjecting the sample to the following atmospheres: (Black) pristine Cu/NiO/Cu(**100**), (red) CO_2_:CO:H_2_ (20:3:1) and (green) CO_2_:H_2_ (20:1) reaction mixture (*p*_T_ = 2.1 mbar). Labels * and ** denote the characteristic oscillations observed in bulk NiO samples. All spectra shown were collected locally from a Cu‐coated NiO island.

Because the spectra were acquired from regions initially occupied by NiO islands, the observed evolution reflects the chemical transformation of these features. The Ni‐L_3,2_ absorption edge retains characteristic NiO features (identified as * and **) throughout most of the reaction but gradually shifts toward a metallic profile after 70 min. However, no changes in the Cu L‐edge reflecting the chemical state of copper were observed in atmospheres of different composition. In the pristine sample, the Cu edge reveals a surface modified by O_ads_, characterized by altered peak ratios near 933 and 937 eV. This is assigned to a surface modification by O_ads_ resulting from the oxidation step responsible for the formation of the NiO islands. Here we discard the formation of Cu_2_O [45], in favor of the adsorption of O in an incipient stage prior to Cu_2_O growth, as also evidenced in LEED. As the sample is subjected to hydrogenation conditions, O is increasingly removed from the surface through its reaction with H_ads_, thus developing a more metallic character in the absorption spectra.

The consistency of the Ni‐L edge across treatments underscores the gradual reduction behavior. One might assume that de‐encapsulation is necessary before NiO reduction can proceed. However, hydrogen, like oxygen, can permeate thin Cu layers due to its low activation barrier for diffusion along the interstitial and grain boundary sites [53, 54]. This supports the possibility of in situ NiO reduction without complete surface exposure. Figure S4 shows locally integrated NEXAFS spectra indicating that, once reduced, Ni remains in the vicinity of the NiO islands and no considerable redistribution across the Cu surface occurs.

Complementary XPS spectra (Figure 2) show averaged signals across the sample. While XPS is not spatially resolved like NEXAFS, the lack of Ni in plane diffusion observed before supports the idea that these data still reflect local behavior. The Cu 2p_3/2_ binding energy remains nearly unchanged with the reaction time and CO presence (932.5 vs. 932.2 eV), matching reported values for metallic Cu [55]. The most significant change is observed in the Ni 2p photoemission line, Figure 2e. In the absence of CO, the nickel signal remains undetectable, as in the pristine state. However, upon CO addition, a progressive increase in the Ni signal is observed with reaction time, suggesting that a restructuring of the Cu shell enables Ni reemergence at the surface. This supports a model where NiO islands are reduced under the Cu overlayer by H_ads_. The reduced Ni likely integrates into or near the Cu shell, with its surface segregation modulated by the gas‐phase composition. The driving force for Ni segregation might be therefore tied to the stabilization of surface species by reaction intermediates. While the present LEEM/XPEEM measurements probe restructuring processes at the mesoscopic scale, the observed evolution necessarily reflects underlying atomic‐scale redistribution processes occurring within the Cu encapsulating shell and at the perimeter of the encapsulated NiO islands. In particular, the adsorbate‐induced segregation trends predicted by DFT suggest that nanoscale fluctuations in the local Cu/Ni composition emerge dynamically depending on the steady‐state adsorbate population imposed by the reaction environment. These redistribution processes are expected to be particularly pronounced near the island perimeter, where local diffusion pathways and reactant accessibility may generate distinct chemical potentials relative to the island interior.

The apparent discrepancy between our findings and those reported by Zegkinoglou et al. [34]. for size selected CuNi NPs may be explained by differences in the nature of intermediates formed under the respective reaction conditions and catalyst architecture. To test this hypothesis, we performed density functional theory (DFT) simulations to calculate the segregation energy of both Cu and Ni under different chemical environments. Our analysis assumes thermodynamic equilibrium, and we approximate the Ni segregation energy at a Cu(**100**) surface as:

(1) $$E_{\mathrm{segr}.}\left[\mathrm{Ni}/\mathrm{Cu}\left(100\right)\right]=E_{\mathrm{Ni}/\mathrm{Cu}\left(100\right)}\;-E_{\mathrm{Cu}\left(100\right)}+E_{\mathrm{Cu}}-E_{\mathrm{Ni}}$$

where ***E***_**Ni**/**Cu**(100)_, ***E***_**Cu**(100)_ are computed from DFT slab models of the Ni‐doped and clean Cu(**100**) surfaces, respectively. To additionally capture the effect of a reactive environment in the following, we simply include specific adsorption within these two surface models (Figure S5). As reference for Cu and Ni species (***E***_**Cu**_ and ***E***_**Ni**_), we use the bulk (fcc) metals. The choice of metallic Ni assumes that NiO has already been reduced under reaction conditions. Using a NiO thermodynamic reservoir instead, however, only lowers ***E***_**segr**._ by a constant offset (reflecting the exothermic NiO + H_2_ → Ni + H_2_O reduction) and does not change the qualitative results (Figure S6). With the above definition, more negative ***E***_**segr**._ means more favorable Ni surface segregation.

Assuming a vacuum background (no surface adsorption), we predict endothermic Ni segregation with ***E***_**segr**._ [**Ni**/**Cu**(100)]  =   + 0.41 eV when specifically exchanging one in nine Cu atoms with Ni in the uppermost surface layer. This prediction follows chemical intuition, with Cu preferentially exposed at the surface as the more noble of the two metals. The preference for Cu is thus founded in the significantly lower Cu(**100**) surface free energy of 208 meV/Å^2^ when compared to the corresponding 227 meV/Å^2^ for Ni(100). The same trend emerges when considering any nonreactive environment, such as CO_2_ gas. DFT predicts a (physisorbed) weakly interacting CO_2_ state above the Cu(**100**) surface ((*)CO_2_) and resulting ***E***_**segr**._ [**Ni**/**Cu**(100)]  =   + 0.39 eV. In agreement with previous work [34], however, the segregation preference is reversed in the presence of more strongly interacting species such as *CO (where * denotes here a chemisorbed configuration). In the case of *CO, and for the same 1/9 ML coverage as above, we predict ***E***_**segr**._ [**Ni**/**Cu**(100)]  =   − 0.52 eV, which suggests a clear thermodynamic driving force to segregate Ni at the surface, in line with the preferred chemisorption of CO on Ni compared to Cu [56]. Taken together, our simple model is consistent with experimental observations that show Cu segregation in a CO_2_ atmosphere and Ni segregation in a CO‐containing atmosphere. The emerging mechanistic picture, therefore is one where surface segregation is driven by the competition of keeping the more noble metal at the surface versus exposing the stronger binding metal to a reactive environment [57, 58]. These observations suggest that the active catalyst surface undergoes a dynamic kinetic cycle in which local structure and composition continuously adapt to changes in the chemical potential imposed by the reaction microenvironment. This behavior falls within the broader framework of chemisorption‐induced surface segregation in bimetallic systems, where adsorbate–metal interactions modify the thermodynamic preference for surface composition under reactive environments [11, 59, 60, 61, 62].

In this context, the gas‐phase reactant composition determines the population of adsorbates at the catalyst surface and thereby shifts the balance between metastable Cu‐rich and Ni‐rich surface terminations. While O‐bound intermediates stabilize Cu termination, strongly C‐bound species such as *CO promote Ni segregation, effectively allowing the reaction microenvironment to steer the abundance and identity of the catalytically active surface sites under operando conditions.

We establish the above understanding by extending our analysis to include not only *CO and (*)CO_2_, but also various other surface‐bound intermediates that may appear along the reaction path during CO/CO_2_ hydrogenation [34]. The results are shown in Figure 5, which plots *E*_segr._ [Ni/Cu(**100**)] as a function of the difference in adsorption energies at a Ni(100) versus Cu(**100**) surface (Δ ***E***_ads_ = ***E***_ads on Ni_  − ***E***_ads on Cu_). First thing that we note is that this Δ ***E***_ads_ descriptor is negative for all adsorbates; a result simply reflecting the fact that Ni is overall a more reactive metal than Cu (Figure S7). The exact value, however, depends upon the type of adsorbate. Interestingly, we consistently find molecular adsorbates that bind to the surface through their C atom to have a more negative Δ ***E***_ads_ as compared to those that bind through their O atom. More negative Δ ***E***_ads_ is generally accompanied by a lower ***E***_segr._ [Ni/Cu(**100**)]. This observation strengthens the previously discussed mechanistic picture: the higher the reactivity toward Ni relative to Cu (lower Δ ***E***_ads_), the stronger the preference for Ni at the surface (lower ***E***_segr._). The only outlier in Figure 5 is *COH which adsorbs at a fourfold hollow site on Ni(100), unlike all other investigated species that adsorb at top or bridge sites. The strong *COH binding through four nearest Ni neighbors predicts a lower energy gain when driving only a single Ni atom to the Cu(**100**) surface (as considered here). Disregarding *COH, however, the remaining data outline an (approximately) linear Δ ***E***_ads_ versus ***E***_segr._ relation in Figure 5. As a result, we find C‐bound species in the lower left part of the plot with ***E***_segr._ < 0, while O‐bound and physisorbed species lie in the upper right part of the plot with ***E***_segr._ > 0. This result is perfectly consistent with the previously predicted inversion in the Cu/Ni surface segregation when switching between (O‐bound) *OCH_3_ and (C‐bound) *CO adsorbates [34]. Elucidating past [10, 34] and present experimental data, our analysis indeed suggests *CO as one of the strongest (thermodynamic) drivers of Ni surface segregation. However, neither CO nor the other investigated C‐binding intermediates are expected to induce significant segregation under the CO_2_ + H_2_ hydrogenation conditions, where their steady‐state surface coverages remain low. Instead, our experimental observations suggest that segregation is only triggered when appreciable CO coverages are established through the presence of CO in the CO_2_ + H_2_+ CO feed gas. Under relevant reaction conditions, however, the segregation profile will ultimately be determined by the ongoing kinetic competition for surface sites.

**FIGURE 5:**
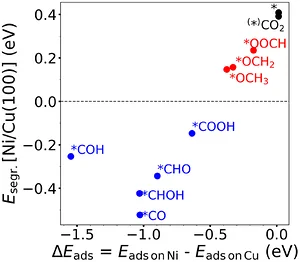
Segregation energy of Ni at Cu(**100**) in the presence of different adsorbates (C‐bound species in blue, O‐bound species in red, physisorbed species, and clean surface (*) in black). The *x*‐axis shows the difference in adsorption energy for each adsorbate on a clean Ni(100) versus a clean Cu(**100**) surface. C‐bound species drive Ni enrichment in the outermost layer (*E*_segr_ < 0), whereas O‐bound species favor Cu termination (*E*_segr_ > 0).

### 2.3 Real Time In Situ Measurements on the NiO Island Dynamics: NAP‐LEEM

We now address the dynamic aspect of the encapsulation/decapsulation process of the NiO particles under reaction conditions. As often criticized in the catalysis community, quasi in situ measurements rely on the assumption that the structures formed under reaction conditions remain unaltered after the reaction environment is removed. Because our aim is to determine how segregation behavior depends on the composition of the reaction atmosphere, it is critical to verify that conclusions drawn from quasi in situ observations also hold true in situ under the reactive environment.

To this end, we carried out real time monitoring of the evolution of Cu‐encapsulated NiO islands supported on Cu(**100**) in a (40:2:3) H_2_+CO_2_+Ar feed using a NAP LEEM setup. While no spectroscopic measurements could be acquired in this configuration (this prototype microscope is not yet connected to a synchrotron beamline), identical reaction conditions to those described above were used.

Figure 6a shows a time series of LEEM images recorded under a CO_2_ hydrogenation reaction mix without CO. As in the quasi in situ experiments, dark features correspond to encapsulated NiO islands. The decrease in island size is directly observed in real time (see Video S1). The shrinking process is heterogeneous: i) “red” islands are the most active, disappearing first; ii) “green” islands shrink more slowly or with an observable delay; and iii) “blue” islands show no significant change within the observation window.

**FIGURE 6:**
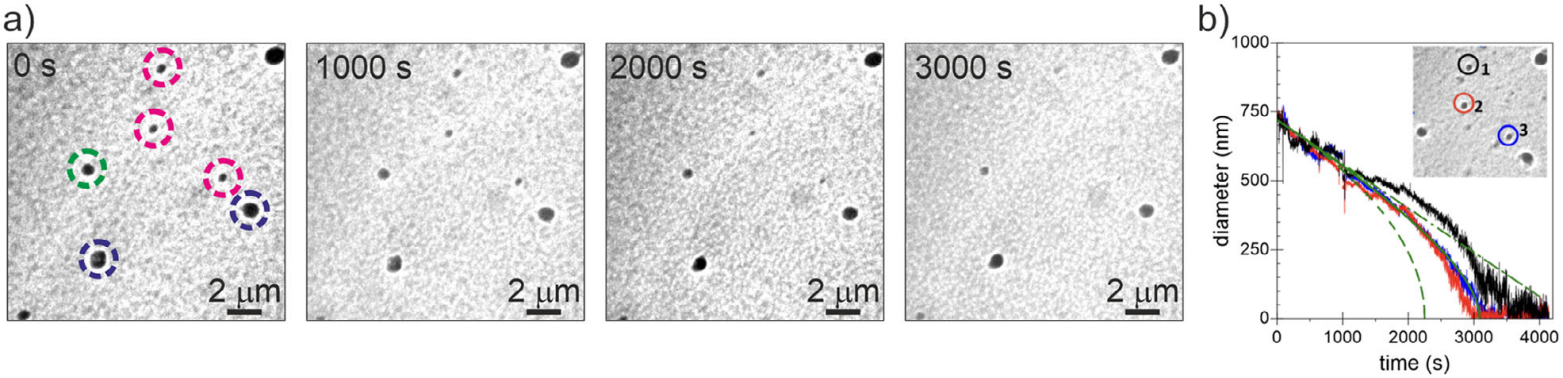
(a) Time series of LEEM images collected at 543 K and an electron energy of 0.5 eV. Dark features correspond to encapsulated NiO islands supported on a Cu(**100**) surface. Reaction conditions: CO_2_ + H_2_ + Ar at a total reaction pressure of 2 × 10^−2^ mbar. $f_{CO_{2}}=0.03\;\mathrm{mL}/\min,\;f_{H_{2}}=0.6\;\mathrm{mL}/\min,\;f_{\mathrm{Ar}}=0.045\;\mathrm{mL}/\min$. *T*_reaction_ = 543 K. (b) Time dependent changes in the diameter of selected NiO islands, as indicated in the inset. Green curves correspond to the fitting results of three different models: (solid line) *D*_H_ = 9 nm^2^/s and *k*_red_ = 0 0.055 nm/s; (dashed line) *D*_H_ = 29 nm^2^/s and (dashed‐dotted line) *k*_red_ = 0.1 nm/s.

To quantify these changes, we tracked the diameter of three selected islands over time (Figure 6b, see details in Supp. Inf. for details). The regular jumps observed in the curves correspond to changes in the focus condition made during the observation.

The size decay was analyzed using an microkinetic model including the following key reaction steps:

(2) $$H_{2}+2*\;⇌2H_{\mathrm{ads}}$$

(3) $$\mathrm{NiO}+\;*\;⇌\mathrm{Ni}+O_{\mathrm{ads}}$$

(4) $$2\;H_{\mathrm{ads}}+O_{\mathrm{ads}}\;⇌H_{2}O_{\mathrm{ads}}+2*$$

(5) $$H_{2}O_{\mathrm{ads}}\;⇌H_{2}O_{\mathrm{gas}}+\;*$$

where * denotes an adsorption site. Scheme 2 illustrates possible locations for these steps. Oxygen is confined to areas containing NiO islands, whereas atomic hydrogen is generated via dissociative adsorption on Cu. Because the NiO islands are encapsulated by Cu (as described in previous sections), H_2_ dissociation within the microkinetic model is described through an effective reduction rate constant (*k*_red_) associated primarily with Cu sites − either on terraces between islands or within the Cu overlayer atop NiO. At the reaction temperature (543 K), a short residence time of dissociated H_2_ on Cu(**100**) is anticipated from TPD data (*T*_desorption_ < 350 K [63]), making its proximity to the island's perimeter as a source for hydrogen important for reduction. Water formed through Equation 4, is also expected to desorb rapidly [64]. During the early stages of the reduction, where the surface remains predominantly Cu‐terminated, H_2_ activation is therefore expected to occur mainly on Cu sites. At later stages, particularly under CO‐containing environments where Ni segregates toward the outermost surface layers of the encapsulating Cu shell, additional H_2_ activation at exposed Ni or Ni–Cu interfacial sites may further contribute to the effective reduction kinetics.

**SCHEME 2:**
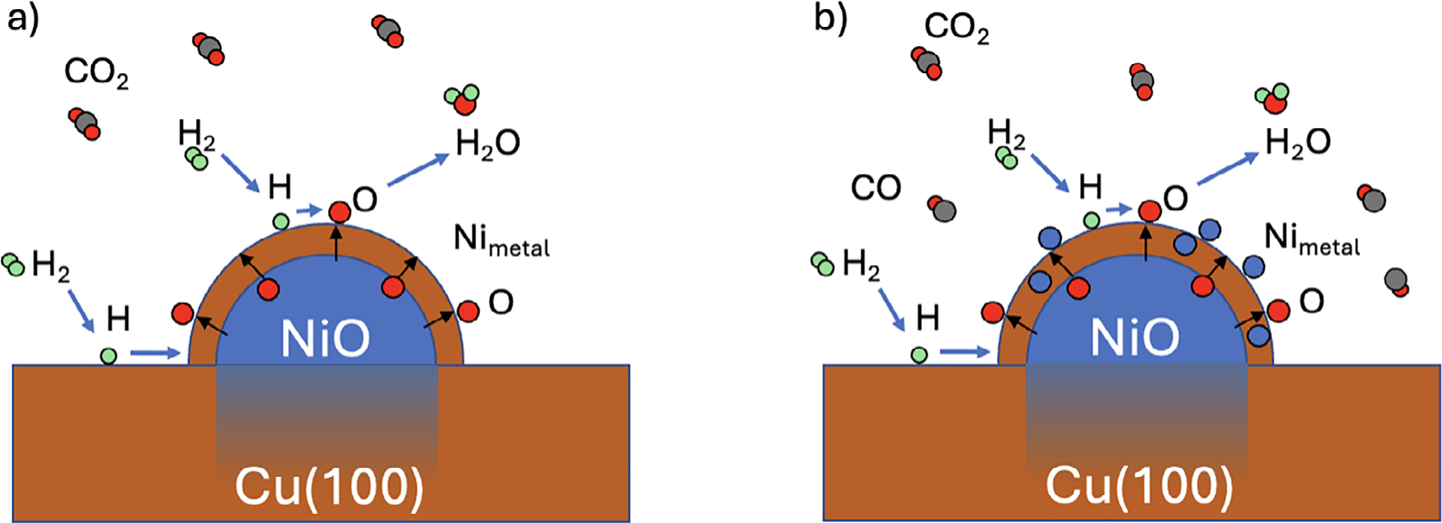
Model depicting the processes occurring on the Cu‐encapsulated NiO island deposited on Cu(**100**) during the reduction step in CO_2_ + H_2_ (a) and CO_2_+CO+H_2_ (b) conditions. Ni atoms are shown in blue, oxygen in red, carbon in gray and hydrogen in green.

Upon NiO reduction, the resulting metallic Ni redistributes according to the reactant mix composition. In the CO‐free conditions, Ni tends to dissolve into subsurface layers of the Cu(**100**) crystal in the vicinity of the islands, whereas in CO‐containing atmospheres, Ni is stabilized at the outermost surface layers of the encapsulating Cu shell. Similarly, intermediates and precursors from CO_2_ hydrogenation, as identified by DFT (Figure 5), will influence the final Ni distribution.

Based on these mechanistic considerations, two parameters describe the change in island size over time: the apparent reduction rate constant (*k_red_*) and the effective diffusion coefficient of H_ads_ to the island perimeter (*D_H_*). A detailed description of the mathematical model is available in the supporting information.

The volume change rate ($\frac{\mathrm{dV}(t)}{\mathrm{dt}}$) can be expressed as:

(6) $$\;\frac{dV\left(t\right)}{dt}=-k_{red}A_{surf}-D_{H}L_{rim}$$

where ***A***_***surf***_ is the encapsulated surface area, ***L***_***rim***_ the island perimeter length, ***k***_***red***_ the apparent reduction rate constant, and ***D***_***H***_ the hydrogen diffusion coefficient. Two limiting cases emerge from the model: i) when ***k***_***red***_ dominates, the diameter decreases linearly with time; ii) when H_ads_ supply at the perimeter dominates, the decrease is a square‐root‐like dependence. Neither extreme fully describes the experimental data (Figure 6b, dashed and dash‐dotted curves), but a combined scenario reproduces the observed curve well, yielding 9 nm^2^/s and 0.055 nm/s for ***D***_***H***_ and ***k***_***red***_. The model also identifies a 400 nm threshold diameter below which the island surrounding area exhibits a significant contribution to reducibility.

Overall, this study delivers a detailed, operando‐level view of how encapsulated NiO islands evolve under CO_2_ hydrogenation conditions, providing insights that extend well beyond the specific Ni–Cu system examined here. By directly visualizing island reduction in real time and linking these observations to gas‐phase composition, we identify clear levers—hydrogen availability, island size, and adsorbate identity—that control the balance between surface segregation and subsurface incorporation of Ni. These findings address a long‐standing challenge in heterogeneous catalysis: disentangling the respective roles of encapsulation, diffusion, and adsorbate‐driven restructuring in determining active‐site composition under working conditions. The methodology demonstrated here—combining spatially resolved spectroscopy, operando microscopy, and atomistic modelling—offers a blueprint for investigating dynamic surface processes in other bimetallic systems and highlights the potential for deliberately tuning catalyst structure and performance through reaction environment design.

## 3 Conclusion

Our work establishes an adsorbate‐controlled segregation principle for Cu–Ni catalysts under CO_2_ hydrogenation conditions using as target system NiO islands encapsulated by a thin (∼3 nm) Cu shell and deposited on Cu(**100**). Upon exposure to CO_2_ hydrogenation conditions, the NiO islands become smaller, with the resulting metallic Ni remaining confined near the original island locations. Interestingly, while the presence of CO in the gas feed does not alter the reducibility of the NiO islands, it markedly changes the post‐reduction distribution of Ni and Cu: CO promotes Ni surface segregation into the Cu shell, lowering the overall surface free energy.

DFT calculations reveal that the reaction environment plays a decisive role in driving segregation: O‐bound adsorbates stabilize Cu on the surface, whereas C‐bound species favor Ni surface enrichment. This adsorbate‐specific stabilization principle provides a framework for predicting operando surface composition in bimetallic catalysts during carbon conversion vs. oxidation catalysis.

Finally, NAP‐LEEM observations provided insight into the complex dynamic transformations taking place in these catalysts in distinct CO_2_ hydrogenation environments. NiO reduction occurs during hydrogenation in the presence and absence of CO in the CO_2_+H_2_ reaction mixture. Real‐time observation in the CO_2_+H_2_ environment indicates that the reduction proceeds heterogeneously, with its kinetics strongly dependent on the island size. Time‐resolved analysis indicates that hydrogen availability on the island surface and perimeter plays an important role during the reduction, suggesting that both, the island geometry and its spatial arrangement could be leveraged to tune catalyst performance. Beyond the specific system investigated here, the combination of operando spectro‐microscopy, with kinetic modelling aided by DFT calculations provides a framework for identifying dynamic descriptors that govern catalyst restructuring under reaction conditions. Parameters such as local hydrogen availability, island‐size‐dependent reducibility, and adsorbate‐driven mass transport can in principle be extracted directly from time‐resolved observations and correlated with changes in the surface composition and active‐site populations. Importantly, the present observations further emphasize that catalytically active sites should not be regarded as static entities, but rather as dynamic surface motifs whose structure and composition continuously adjust in response to changes in the local chemical potential driven by the reaction microenvironment. Such restructuring processes therefore need to be considered explicitly in future theoretical descriptions of catalytic function under operando conditions. While realistic catalysts exhibit significantly greater structural complexity, including multiple facets, defects, interfaces, and more heterogeneous particle‐size distributions, the competing driving forces identified here are expected to operate locally in practical catalytic materials as well. Ultimately, such approaches may enable predictive catalyst design strategies in which reaction microenvironments are deliberately engineered to stabilize desired active site populations, surface states, and restructuring pathways under operando conditions.

## Author Contributions

**Mauricio J. Prieto**: conceptualization, investigation, writing – original draft, writing – review and editing, formal analysis, data curation, methodology, validation, visualization, project administration. **Hao Wan**: investigation, writing – original draft, formal analysis, data curation, validation, visualization, writing – review and editing. **Liviu C. Tănase**: investigation, methodology, writing – review and editing. **Lucas de Souza Caldas**: methodology, investigation, writing – review and editing. **Aarti Tiwari**: investigation, methodology, writing – review and editing. **Karsten Reuter**: supervision, writing – review and editing, conceptualization, funding acquisition. **Hendrik H. Heenen**: conceptualization, investigation, writing – original draft, writing – review and editing, methodology, formal analysis, supervision, validation, visualization. **Vanessa J. Bukas**: conceptualization, investigation, writing – original draft, writing – review and editing, methodology, formal analysis, supervision, validation, visualization. **Thomas Schmidt**: supervision, conceptualization, investigation, writing – original draft, writing – review and editing, validation, visualization, methodology, project administration, formal analysis. **Beatriz Roldan Cuenya**: conceptualization, investigation, funding acquisition, validation, writing – review and editing, supervision, resources, visualization, project administration.

## Conflicts of Interest

The authors declare no conflicts of interest.

## Supporting information



Supporting Information file providing complementary experimental and DFT results, as well as the equations used to model and fit the time‐dependent changes in the diameters of selected NiO islands during real‐time measurements. The following video files are provided: (1) Video collected at 820 K in 5 ×10^−6^ mbar O_2_ showing the diffusion of steps on a Cu/NiO/Cu(**100**) surface. (2) Video showing the chemical reduction of NiO islands under CO_2_ hydrogenation conditions. Data collected in real time under the following reaction conditions: CO_2_ + H_2_ + Ar (2:40:3) at a total pressure of 2×10^−2^ mbar. $f_{CO_{2}}=0.03\;\mathrm{mL}/\min,\;\;f_{H_{2}}=0.6\;\mathrm{mL}/\min,\;f_{\mathrm{Ar}}=0.045\;\mathrm{mL}/\min$. T_reaction_ = 543 K.
**Supporting File 1**: anie73988‐sup‐0001‐SuppMat.pdf.

**Supporting File 2**: anie73988‐sup‐0002‐VideoS1.mp4.

**Supporting File 3**: anie73988‐sup‐0003‐VideoS2.mp4.

## Data Availability

The data that support the findings of this study are available from the corresponding author upon reasonable request.
