# Supplementary material for: Structural and Chemical Dynamics of Cu/NiO/Cu(100) Surfaces During CO2 Hydrogenation
Source: Angew Chem Int Ed Engl. 2026 Sep 8;65(44):e8933772. doi: 10.1002/anie.8933772 (PMC13703909; doi:10.1002/anie.8933772)
Supplement: Supplementary file 1 [file ANIE-65-e8933772-s002.pdf]

Supporting Information

Structural and Chemical Dynamics of Cu/NiO/Cu(100) Surfaces during CO_2 Hydrogenation

Mauricio J. Prieto*[a], Hao Wan[b], Liviu C. Tănase[a], Lucas de Souza Caldas[a], Aarti Tiwari[a], Karsten Reuter[b], Hendrik H. Heenen[b], Vanessa j. Bukas[b], Thomas Schmidt[a] and Beatriz Roldan Cuenya*[a]

[a] *Department of Interface Science, Fritz-Haber-Institut der Max-Planck-Gesellschaft, Faradayweg 4-6, 14195 Berlin, Germany*

[b] *Theory Department, Fritz-Haber-Institut der Max-Planck-Gesellschaft, Faradayweg 4-6, 14195 Berlin, Germany*

**prieto@fhi-berlin.mpg.de; roldan@fhi-berlin.mpg.de*

Experimental Section

The experiments were carried out in the SMART microscope operating at the UE49-PGM beamline of the synchrotron light source BESSY II of the Helmholtz Center Berlin (HZB). The aberration corrected and energy filtered LEEM/XPEEM instrument combines microscopy (LEEM/XPEEM), diffraction (μ-LEED), and spectroscopy (μ-XPS) techniques for comprehensive characterization. The base pressure of the system is 10^{-10} mbar, but operation is possible at pressures up to 10^{-5} mbar of reactive gases in a temperature range between 150 and 1500 K.[37-39]

The Cu(100) single crystal was prepared by cycles of Ar^+ sputtering at room temperature and annealing in UHV or oxygen at 870 K, depending on its cleanliness state. Cleaning cycles were repeated until no contamination could be detected by XPS, with terraces a few 100 nm wide and a sharp (1×1) LEED pattern. Sample temperature was measured either by a W26%Re/W5%Re thermocouple or by a pyrometer (IMPAC IGA 740) with an absolute accuracy of ~10 K. O_2 (99.999%), H_2 (99.999%), CO_2 (99.995%) and CO (99.97%) were dosed directly into the experimental chamber; Ni was sublimated from a 4 mm thick rod (99.999%) using a commercial evaporator (Omicron EFM3) under grazing incidence of 20°.

In situ measurements were performed in a near-ambient-pressure (NAP) LEEM/XPEEM microscope. The microscope is not yet connected to a beamline, but it is planned for the future. For these measurements, the NiO@Cu/Cu(100) samples were grown at the SMART setup following established protocols. After their full characterization, the samples were transferred into the NAP-LEEM setup. To desorb and remove any potential C-containing species adsorbed during the transfer step, a refreshing treatment in 5×10^{-6} mbar O_2 at 843 K was performed. CO_2 hydrogenation experiments were performed at a sample temperature of 543 K and at a total pressure of 2×10^{-2} mbar. The reaction mixture consisted of a CO_2+H_2+Ar in the ratio of (2:40:3). Ar was added to the mixture as a balance and controlled gas.

Computational methods

DFT calculations were carried out using the GPAW code[40] which is based on a projected augmented wave method (all-electron frozen core approximation) and solved in real space. The revised Perdew-Burke-Ernzerhof (RPBE)[41] exchange-correlation functional was used and wavefunctions were represented on a uniform real-spaced grid with 0.18 Å grid-spacing. The smearing of the occupation numbers is controlled using the Fermi-Dirac method with a width of 0.1 eV. Default electronic convergence criteria were applied (0.5 meV in total energy, 0.0001 of valence electron density, and Kohn-Sham residuals < 4.0×10^{-8} eV^2 per valence electron). We used the Atomic Simulation Environment (ASE)[42] (in which GPAW is natively integrated) to perform geometry optimizations and data post-processing. To model the Cu(100) and Ni-doped Cu(100) surfaces, periodic slabs with a (3×3) surface unit cell were considered, containing six atomic layers and a vacuum of at least 10 Å was introduced along the surface normal to separate periodic images. All atoms in the top four layers of the slab and the adsorbates are fully relaxed, while the bottom two layers are constrained at the bulk geometry. For the Ni(100) surface, the same setup was used but we limited the system to four atomic layers with the bottom two atomic layers fixed since no segregation energies needed to be computed. Monkhorst-Pack k-points sampling[43] of 3 × 3 × 1 were used for all calculations. In model systems where Ni is present, spin-polarized calculations were performed. The quasi-Newton minimization scheme was employed for the geometry optimizations and systems were relaxed until the forces were less than 0.05 eV/Å.

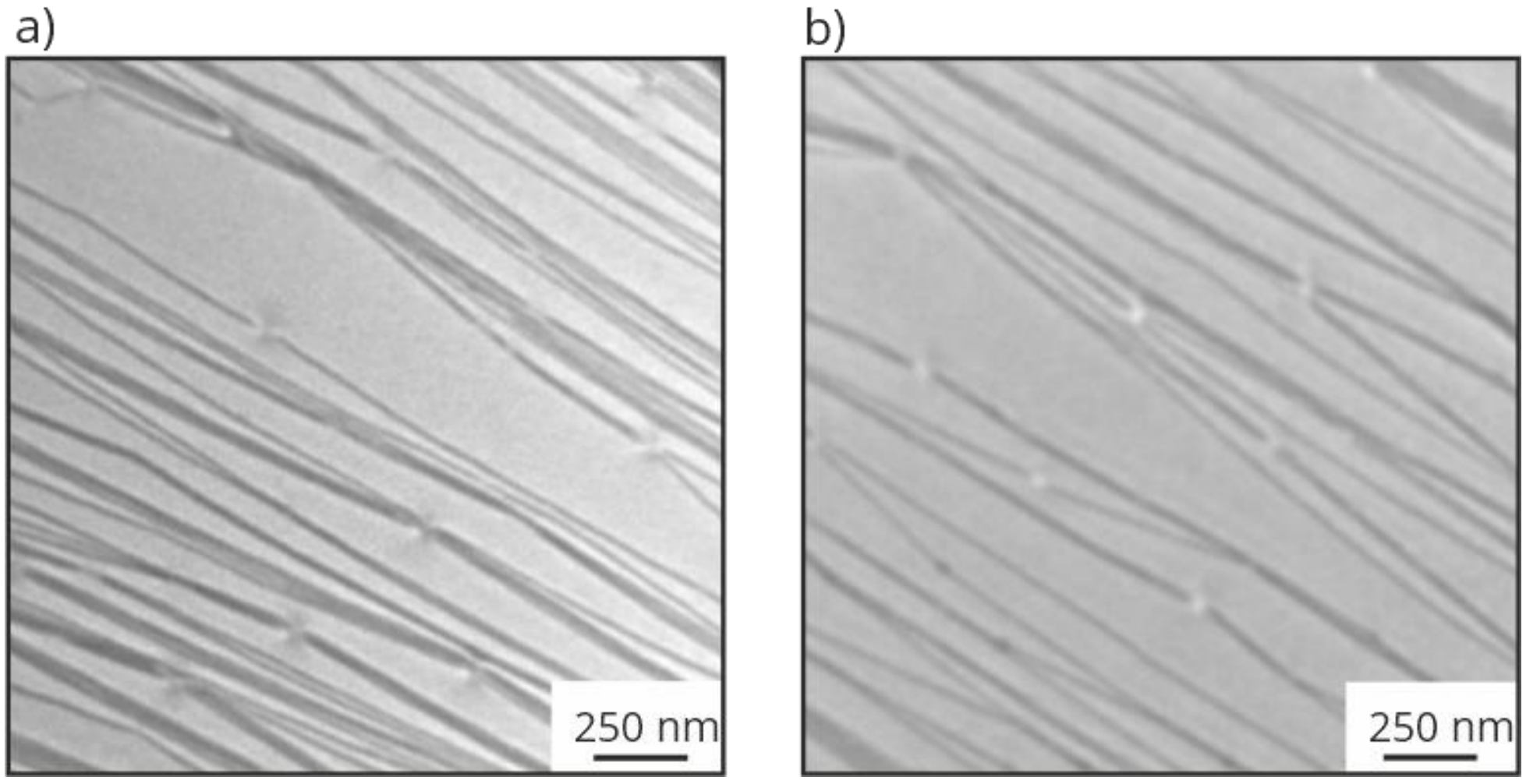

Figure S1. *LEEM images showing (a) pristine Cu(100) and (b) 2 ML of Ni/Cu(100) prepared in UHV conditions. Ni deposition was performed in UHV conditions and at room temperature. Images collected with an electron energy of 5 eV.*

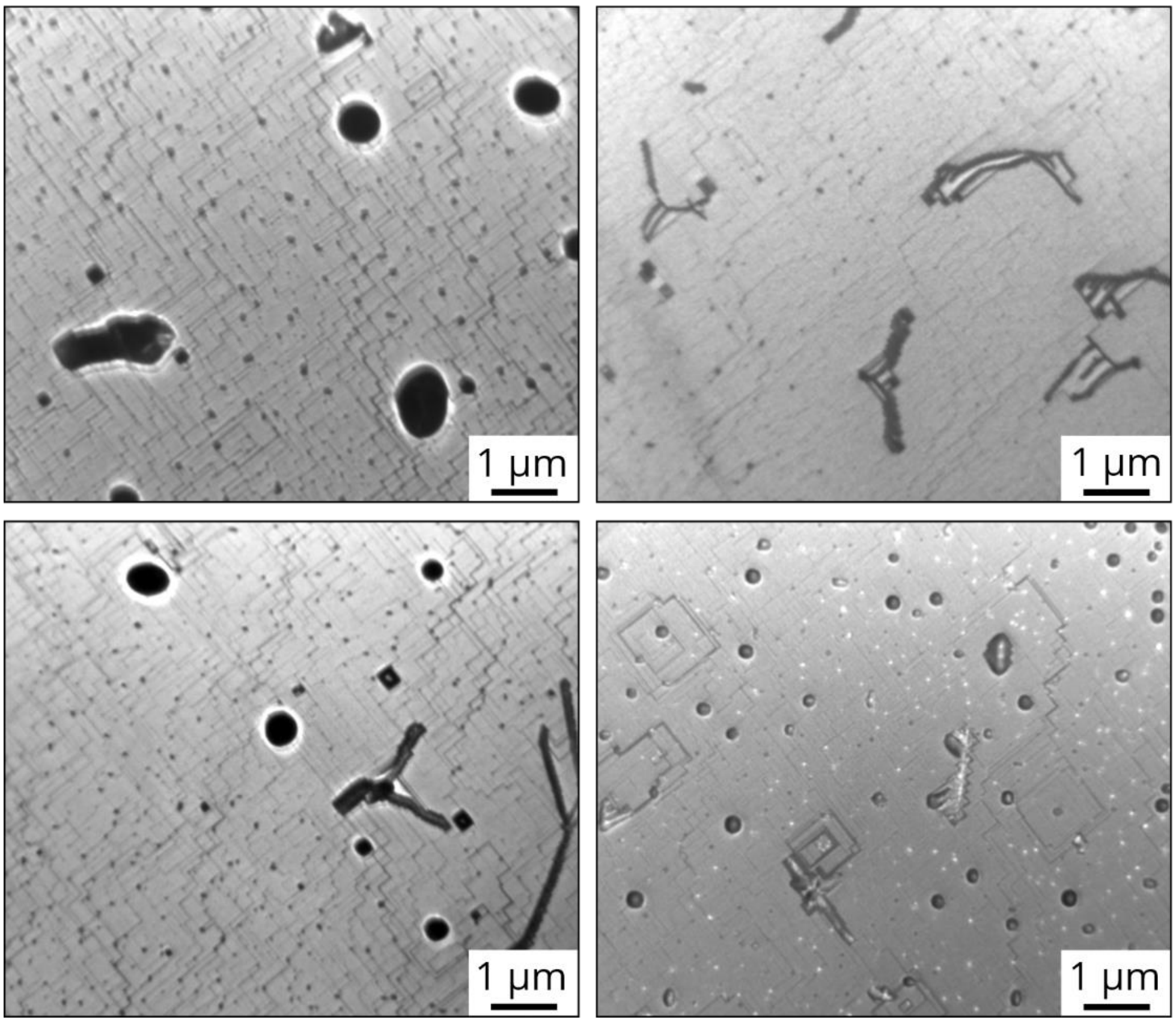

Figure S2. *LEEM images showing the heterogeneity of the structures formed by NiO islands on Cu(100), resulting from the annealing at 820 K in 5×10^{-6} mbar O_2. For all images an electron energy of 5 eV was used. Images were collected in UHV and at room temperature.*

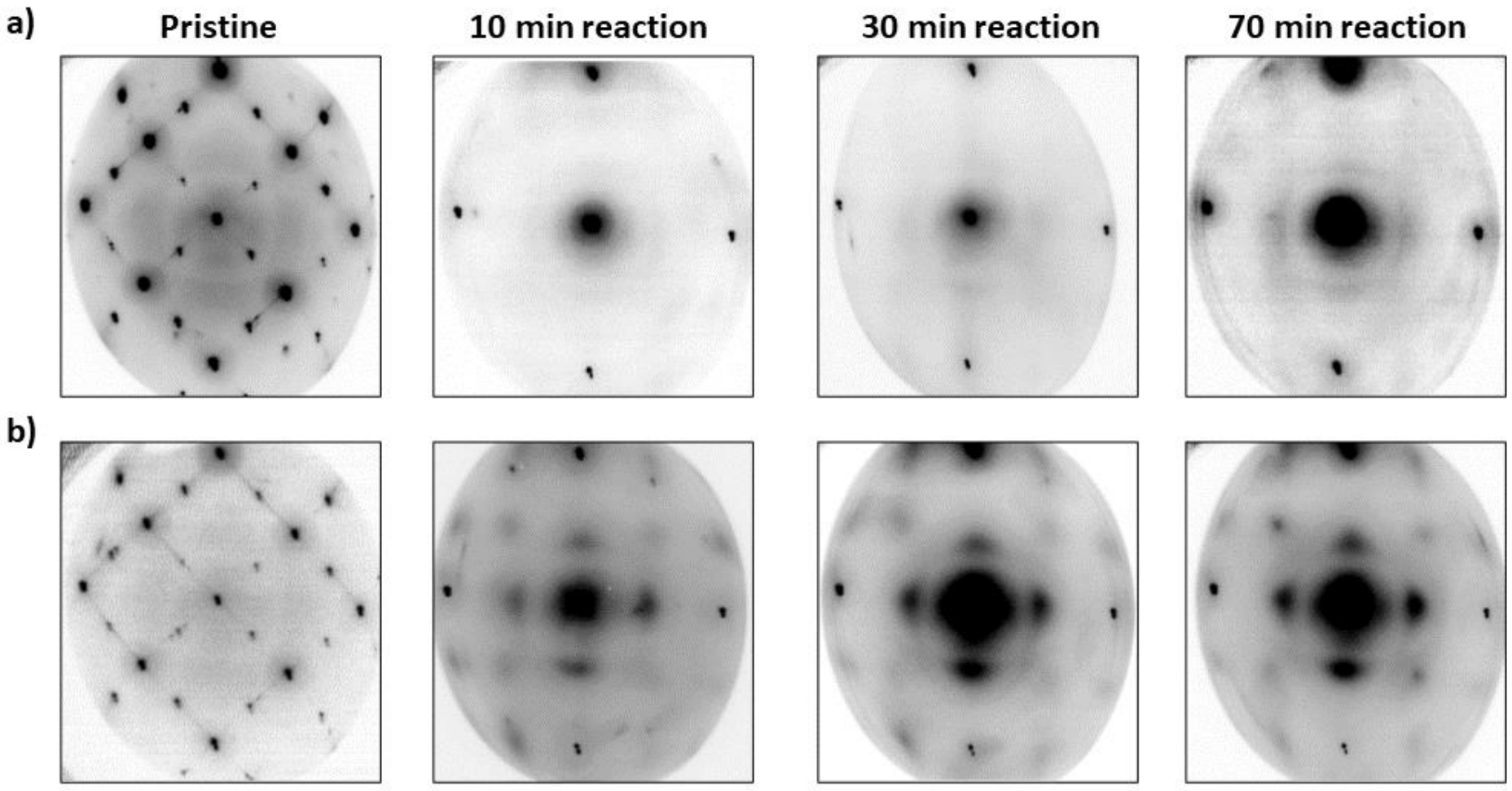

Figure S3. *LEED patterns collected at different stages of sample exposure to a CO_2 hydrogenation environment, as indicated. Reaction performed at 573 K in a) (1:20) CO_2+H_2 reactant mixture (p_T = 2.1 mbar) and b) (1:3:20) CO_2 + CO + H_2 mixture (p_T = 2.4 mbar). All patterns were recorded with an electron energy of 42 eV. The pristine surface shows a* $(2\sqrt{2} \times \sqrt{2})R45°$ *and a diffuse* $p(2 \times 2)$ for the reaction mixture including CO.

Table S1. *Binding energy positions of Cu and Ni $2p_{3/2}$ photoemission lines for the different sample stages. Lines were collected with a photon energy of 1030 and 940 eV, respectively, thus corresponding to electrons with an estimated inelastic mean free path of 4.7 ± 0.2 Å.*

Line	***(1:20) CO_2+H_2 (p_T= 2.1 mbar)***		
	Pristine	10 min reaction	70 min reaction
Cu $2p_{3/2}$	932.2	932.2	932.8
Ni $2p_{3/2}$	n/d	n/d	n/d
	(3:1:20) CO + CO_2 + H_2 (p_T= 2.4 mbar)		
	Pristine	10 min reaction	70 min reaction
Cu $2p_{3/2}$	931.8	932	932
Ni $2p_{3/2}$	n/d	852.1	852.1

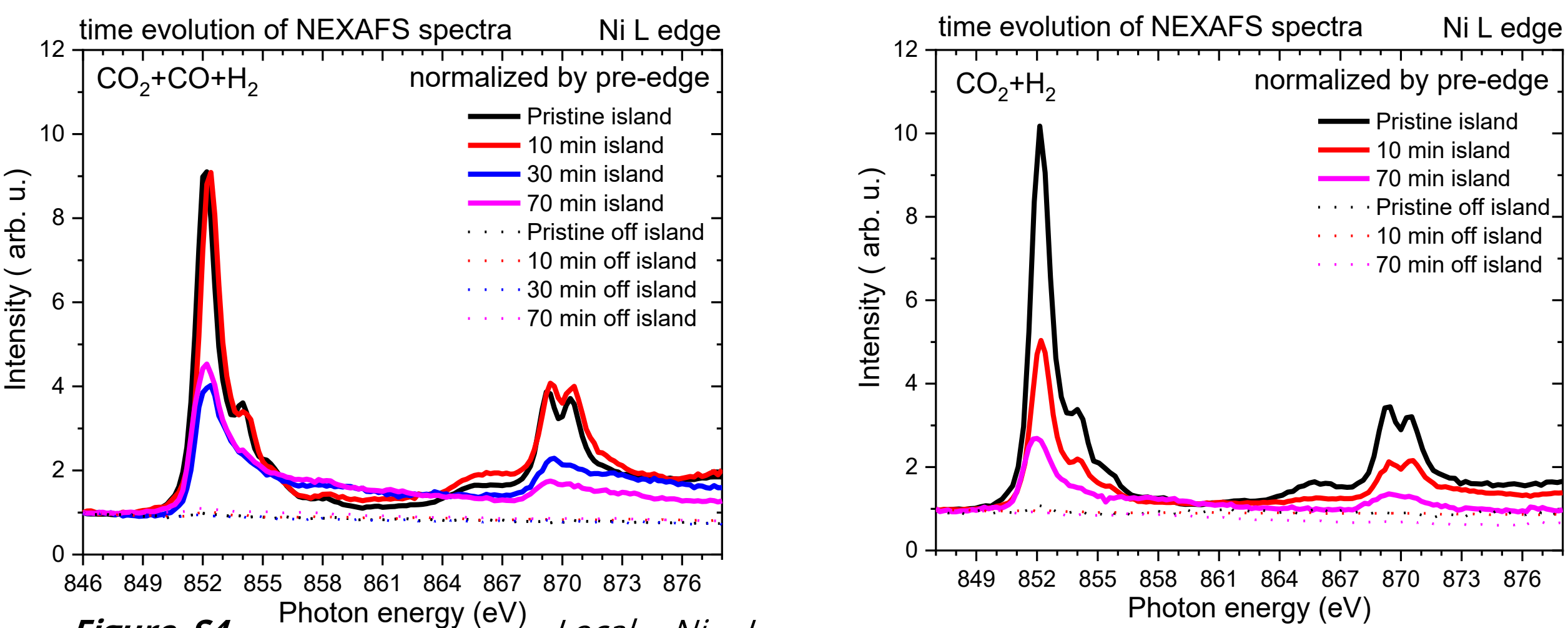

Figure S4. *Local Ni $L_{3,2}$ NEXAFS spectra collected at different stages of CO_2 hydrogenation conditions. Consecutive*

accumulative reaction times, as indicated. Reaction performed at 270 °C in in the presence and absence of CO, as indicated. Composition of the gas phase are (1:3:20) CO_2 + CO + H_2 (p_T = 2.4 mbar) and (1:20) CO_2 + H_2 (p_T = 2.1 mbar)

Density functional simulations

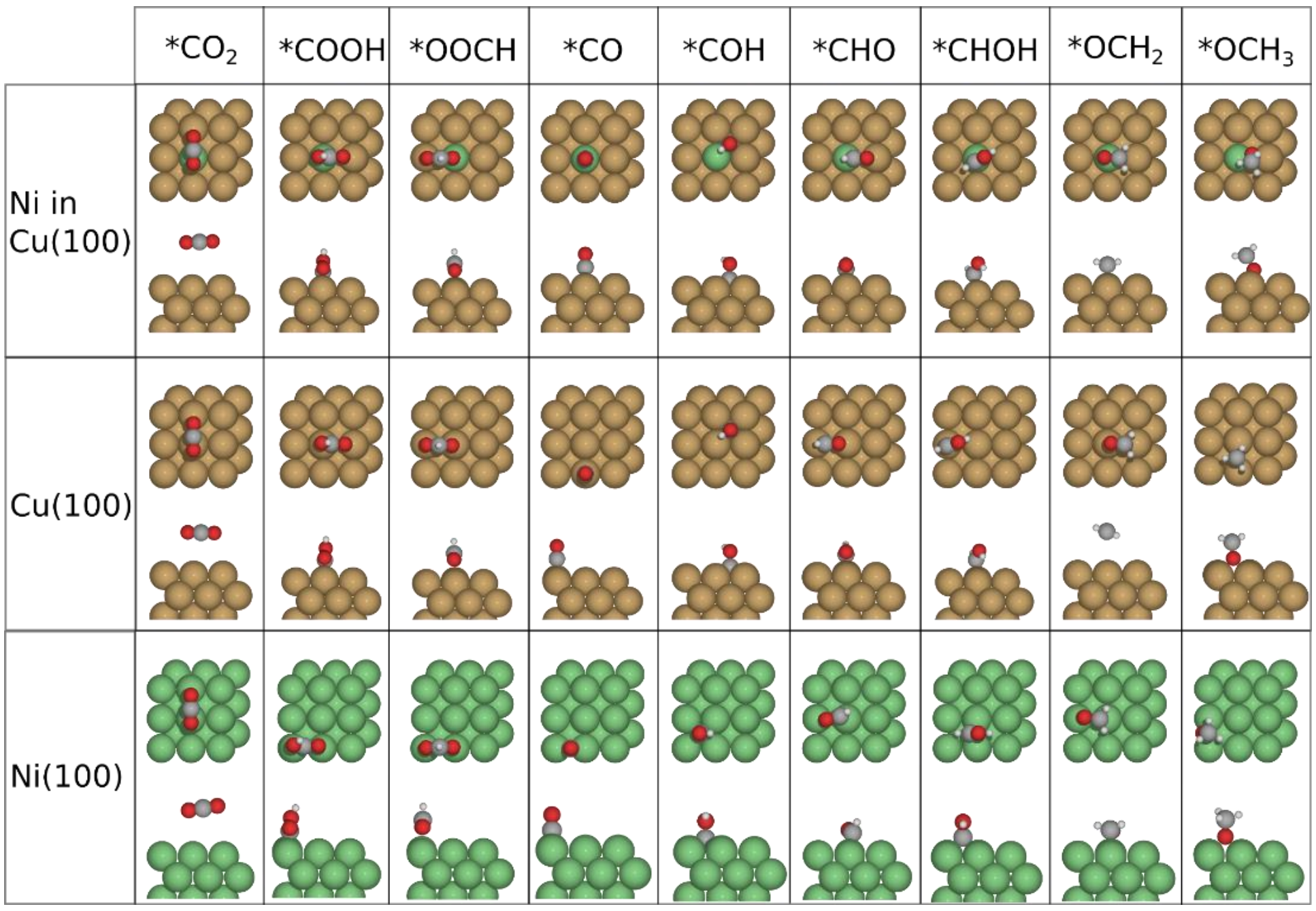

Figure S5. *Top and side views of the (optimized) DFT structures modelling different adsorbates at the Ni-doped Cu(100) surface (top row), clean Cu(100) surface (middle row), and clean Ni(100) surface (bottom row). Atom color-coding is as follows: Cu: gold; Ni: green; C: gray; O: red; H: white.*

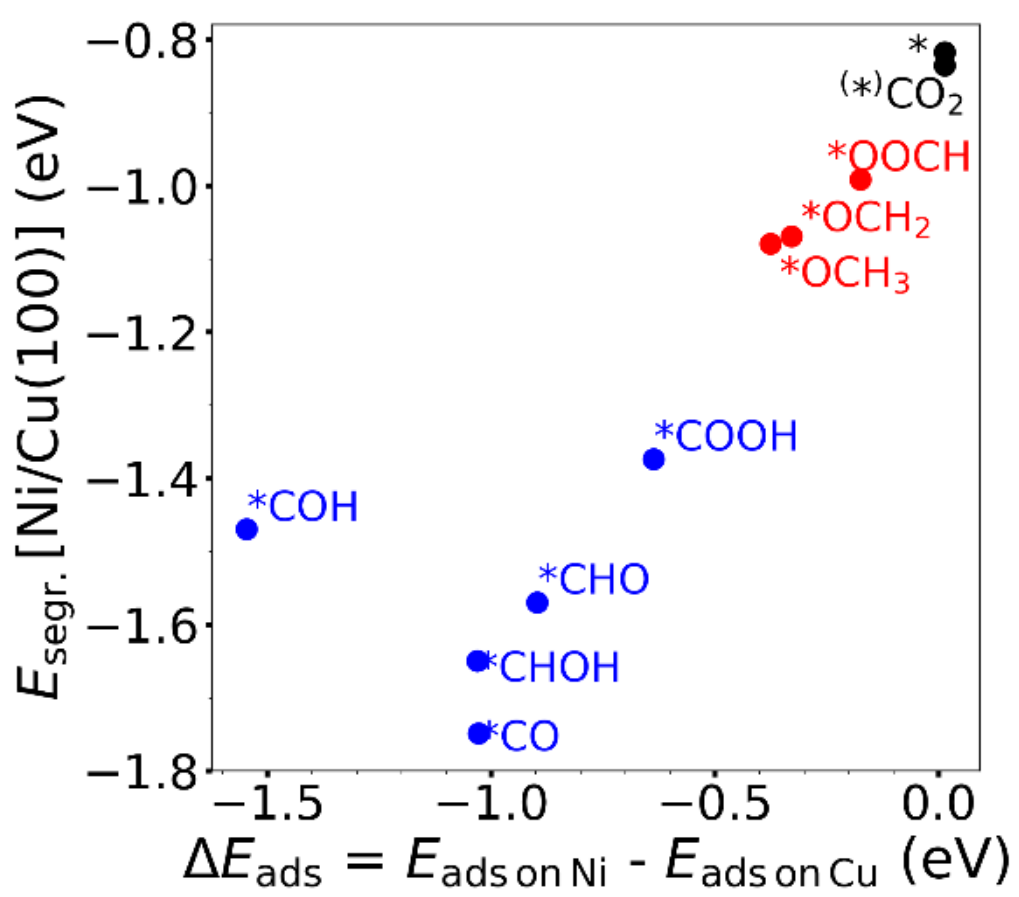

Figure S6. *Same as Fig. 5 of the main text, but assuming bulk (fcc) NiO (rather than metallic Ni) as thermodynamic reservoir for Ni species. The surface segregation energy is therefore calculated as:* $E_{segr.}[Ni/Cu(100)] = E_{Ni/Cu(100)} - E_{Cu(100)} + E_{H_2O} + E_{Cu} - E_{NiO} - E_{H_2}$, *where* E_{H_2O} *and* E_{H_2} *are the DFT energies of gas phase* H_2O *and* H_2 *molecules, respectively.*

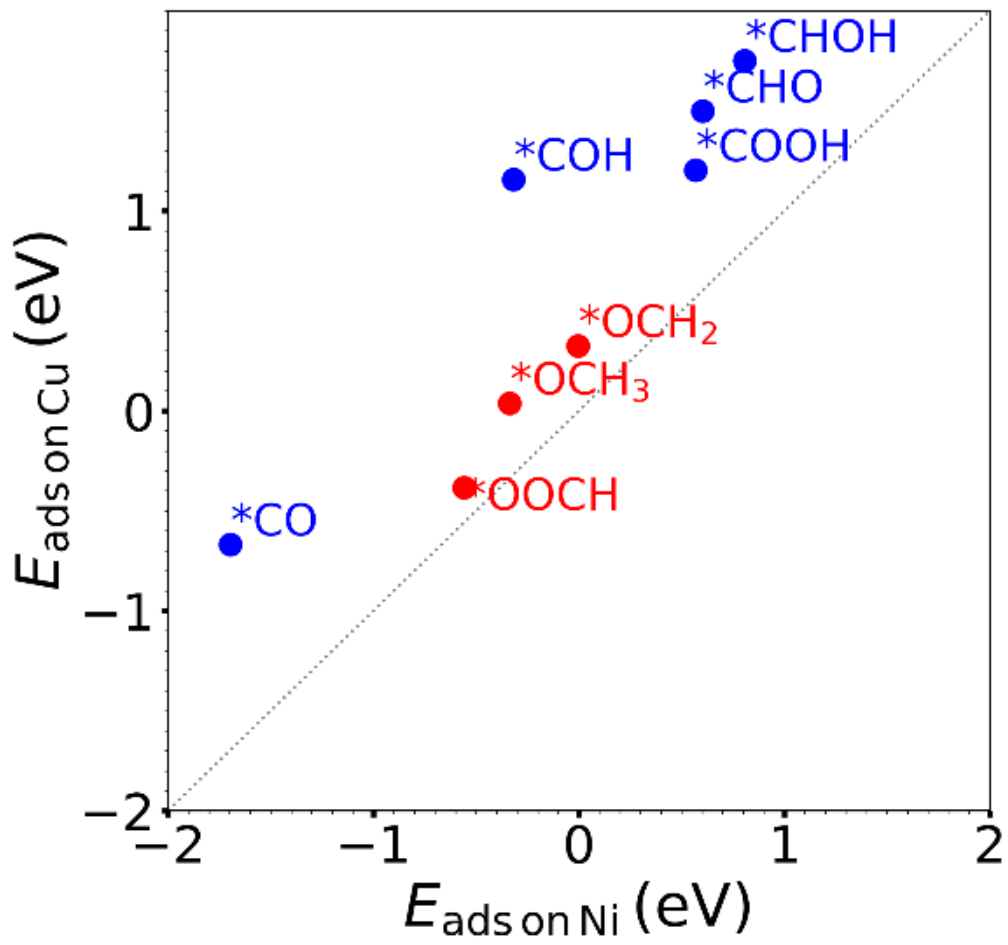

Figure S7. *Comparison of theoretical adsorption energies at (clean) Cu(100) vs. Ni(100) surfaces. C-bound and O-bound adsorbates are shown in blue and red, respectively. The parity line (dotted black) is simply meant to guide the eye. The calculation of adsorption energies uses gas phase CO_2, H_2, and H_2O molecules as reference.*

Time dependence analysis of the NiO island size under reaction conditions

The profile of selected NiO islands was measured as a function of time, based on the dataset of the video made available as supplementary material. In the recorded LEEM images, the islands appear as dark objects (Fig. 6). In order to obtain the island area, a filter was applied: image pixels below a certain level were set to 1 (identifying an island), the rest was set to 0 (identifying the surrounding). In this way, the area size A of an individual island was determined first in pixel and after calibration in nm². Assuming a circular shape, the diameter d was calculated via $d = 2\sqrt{A/\pi}$

The basic idea for the shrinking NiO island is that Ni in the oxidized state is stable, but if it is reduced to the metallic state then Ni can migrate to the substrate interface and can then be dissolved into the Cu bulk. NiO is reduced to Ni by reaction with hydrogen. For this process, various kinetic steps are involved, e.g.:

a) the (dissociative) adsorption of H_2 on the island or surrounding of the island.
b) surface diffusion of H on the surrounding surface and on the island surface.
c) diffusion of O through the bulk of NiO islands.
d) diffusion of Ni through the bulk of NiO islands.
e) diffusion of Ni through the interface of NiO island and Cu support.
f) diffusion of Ni in Cu bulk.
g) dissociation of NiO to Ni and O.
h) formation of H_2O by reaction of H with the O.
i) desorption of H_2O from the surface.

In the following we assume a half sphere shape of the NiO islands with the radius R. The shrinking of the island volume $V(t) = \frac{2\pi}{3}R^3$ might depend on the radius R following a rate equation:

$$\frac{d\,V(t)}{dt} = -\,\beta\,R^n \qquad \text{eq. (1)}$$

The power n is related to the dimension of the dependence and β is the proportionality factor describing the kind of the rate limiting step and the chemical reaction. For each dimension, the rate equation can be easily solved. The results are shown in the following table together with a suggested meaning for the rate limiting step.

n	Rate equation	solution	Meaning for the rate limiting step
0	$\frac{d\,V(t)}{dt} = -\,k_{red}$	$R(t) = \sqrt[3]{{R_0}^3 - \frac{3}{2\pi} k_{red} t}$	Reduction rate independent of the island volume. This can be caused by a single defect in the island
1	$\frac{dV(t)}{dt} = -D_H L_{rim}$	$R(t) = \sqrt{{R_0}^2 - 2D_H t}$	Rate depends on the perimeter. This can be related to the amount of H_2 adsorbed on the surrounding and diffusing through the rim of the island.
2	$\frac{dV(t)}{dt} = -k_{red} A_{surf}$	$R(t) = R_0 - k_{red}\, t$	Rate depends on the surface area of the island. The reaction process on the surface is the rate limiting step
2*	$\frac{dV(t)}{dt} = -D'_{int} A_{int}$	$R(t) = R_0 - \frac{D_{int}}{2} t$	Dependence on interface area between the island and substrate. Rate determined by diffusion of Ni through this border
3	$\frac{dV(t)}{dt} = -k_{red} V$	$R(t) = R_0 e^{-1/3 k_{red} t}$	Reduction rate depends directly on the volume. The reduction process occurs everywhere and arbitrarily in the island bulk

A graphical display for the resulting radius dependences is shown in the following figure for the different dimensions, whereas the initial radius R_0 and the initial temporal change R'_0 are set to be the same.

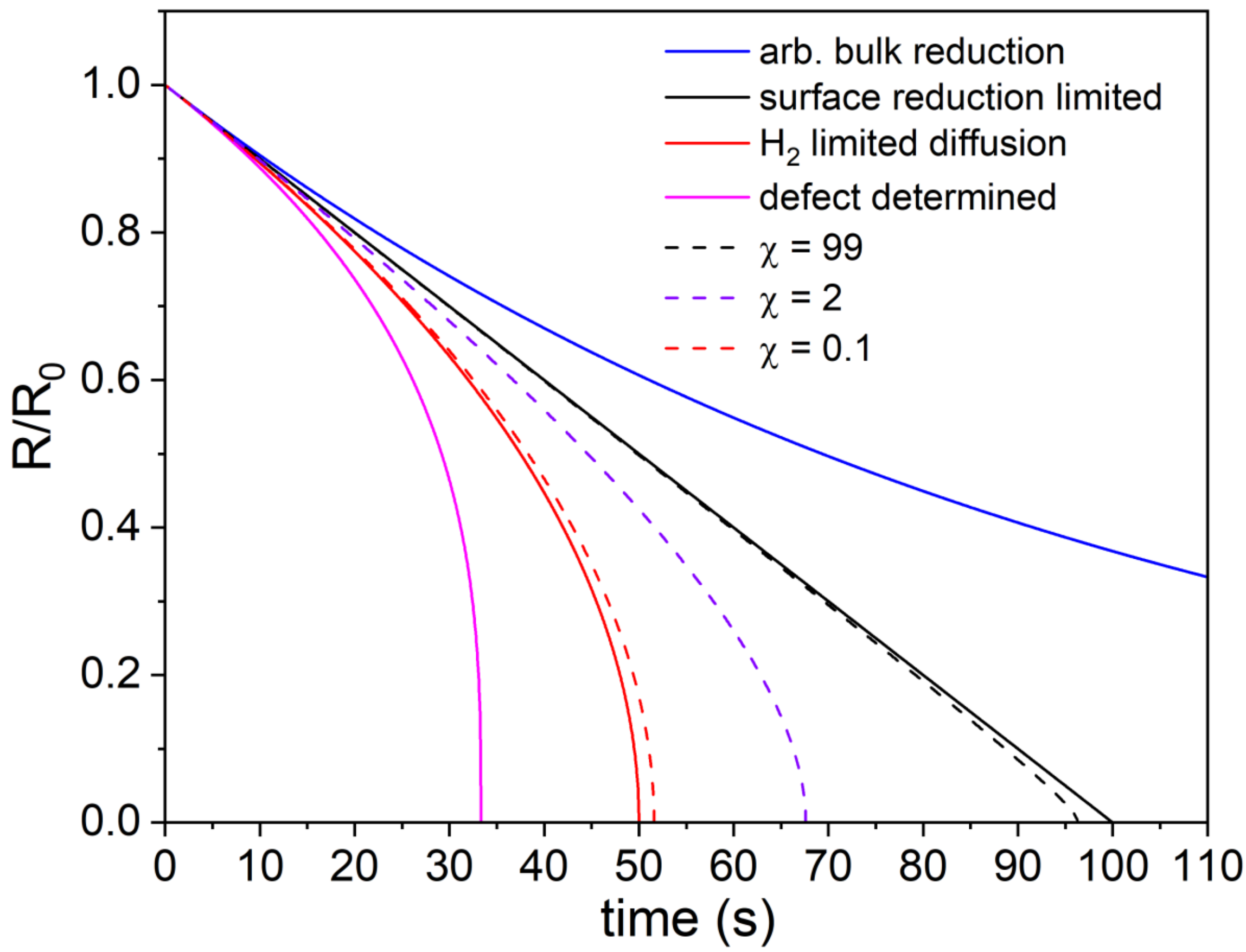

Figure S8: island shrinking for different models

Interestingly, from the initial size R_0 and the time t_0 when the island is completely decayed, one can extract the rate limiting kinetic parameter using the following formulas for the different dimensions:

n	t_0 time for decay	R'$_0$	remarks
0	$t_0 = \frac{{R_0}^3}{\gamma k_{red}} = -\frac{1}{3}\frac{R_0}{R_0'}$	$R_0' = -\frac{\gamma k_{red}}{3{R_0}^2}$	$\gamma = \frac{3}{2\pi}$, $[k_{red}] = \frac{\mathrm{m}^3}{\mathrm{s}}$
1	$t_0 = \frac{{R_0}^2}{2D_H} = -\frac{1}{2}\frac{R_0}{R_0'}$	$R_0' = -\frac{D_H}{R_0}$	$[D_H] = \frac{\mathrm{m}^2}{\mathrm{s}}$
2	$t_0 = \frac{R_0}{k_{red}} = -\frac{R_0}{R_0'}$	$R_0' = -k_{red}$	$[k_{red}] = \frac{\mathrm{m}}{\mathrm{s}}$
2*	$t_0 = \frac{2R_0}{D_{int}} = -\frac{R_0}{R_0'}$	$R_0' = -D_{int}/2$	$[D_{int}] = \frac{\mathrm{m}}{\mathrm{s}}$
3	$t_0 = \infty$	$R_0' = -{}^1\!/_3\, k_{red} R_0$	$[k_{red}] = \frac{1}{\mathrm{s}}$

Our experimental data can only be described by the combination of two of the above models, namely the diffusion across the rim (n=1) and a surface process (n=2):

$$\frac{dV(t)}{dt} = -k_{red}A_{surf} - D_H L_{rim} \qquad \text{(eq. 2)}$$

In contrast to the simple rate equations from above (eq. 1), a direct analytical solution for $R(t)$ does not exist. However, one finds easily the solution for the inverse function $t(R)$, meaning the

shrinking time t to reach a radius R. This inverse function was used to fit our experimental data and to extract the kinetic parameters.

Solution path:
After substituting the volume $V(t) = \frac{2}{3}\pi R^3$, the surface area $A_{surf}(t) = 2\pi R^2$ and the rim length $L_{rim}(t) = 2\pi R$ we get:

$$\frac{dR^3}{dt} = -3\,k_{red}\,R^2 - 3D_H R$$

With $\frac{dR^3}{dt} = 3R^2\frac{dR}{dt}$ and dividing by 3:

$$R\frac{dR}{dt} = -k_{red}R - D_H \qquad (eq.2')$$

and division by the right side, we have the following form:

$$-\frac{R}{k_{red}R + D_H}dR = dt$$

A general solution is:

$$\frac{D_H\ln(k_{red}R + D_H)}{k_{red}^2} - \frac{R}{k_{red}} = t + C \qquad (\text{eq. }3)$$

C is a constant value depending on the initial radius $R_0 = R(t = 0)$. Setting t=0, we get:

$$\frac{D_H\ln(k_{red}R_0 + D_H)}{k_{red}^2} - \frac{R_0}{k_{red}} = C$$

Inserting this into (eq. 3) reads:

$$\frac{D_H}{k_{red}^2}\ln\left(\frac{k_{red}R + D_H}{k_{red}R_0 + D_H}\right) - \frac{(R - R_0)}{k_{red}} = t \qquad (eq.4)$$

This inverse function $t = t\,(R)$ is used to fit our data.
To calculate the time t_0 for the full decay, we set R=0 and get:

$$t_0 = \frac{D_H}{k_{red}^2}\ln\left(\frac{D_H}{k_{red}R_0 + D_H}\right) + \frac{R_0}{k_{red}} \qquad (eq.5)$$

Using (eq. 2′) and inserting the initial radius R_0 at $t=0$, we yield the initial change of the radius:

$$R_0' = -k_{red} - \frac{D_H}{R_0} \qquad (eq.6)$$

The structure of the formula can be simplified by this substitution:

$$\chi = \frac{k_{red}R_0}{D_H} \qquad (eq.7)$$

Whereas χ is dimensionless because of the units $[k_{red}] = \frac{\text{m}}{\text{s}}$, $[D_H] = \frac{\text{m}^2}{\text{s}}$ and $[R] = m$. Now eqs. 4 to 6 read:

$$t = -\frac{R_0}{R_0'}\frac{1+\chi}{\chi}\left(\frac{\ln\left(\frac{1+\chi\frac{R}{R_0}}{1+\chi}\right)}{\chi} + \left(1 - \frac{R}{R_0}\right)\right) \qquad (eq.8)$$

$$t_0 = -\frac{R_0}{R_0'}\frac{1+\chi}{\chi}\left(1 - \frac{\ln(1+\chi)}{\chi}\right) \qquad (eq.9)$$

$$R_0' = -k_{red}\frac{1+\chi}{\chi} \qquad (eq.10)$$

It is worth to mention that the curve of eq. 4 (eq. 8) fits just between the corresponding curves of n = 1 and n = 2 (see dashed curves in Fig. S8). Also, if one set either $D_H \to 0$ or $k_{red} \to 0$ one gets the

formula for the simple cases (n = 1 or n = 2). Also, we get from eq. 5 or 8 the values $t_0 = \frac{R_0}{k_{red}}$ for $D_H \to 0$ and $t_0 = \frac{R_0{}^2}{2D_H}$ for $k_{red} \to 0$. For this, one has to apply the L'Hôpital's rule.[44]

This last model combines the rate limitation of the NiO reduction at the island surface with the limitation by the H_2 diffusion from the surrounding. Which of the two processes is the dominating one might change during the shrinking. This is described by a critical radius R_{crit}:

$$R_{crit} = \frac{D_H}{k_{red}} = \frac{R_0}{\chi} \qquad (eq. 11)$$

For $R > R_{crit}$ the shrinking is dominated by the reduction on the island surface (i.e. $k_{red}A_{surf} > D_H L_{rim}$) whereas for $R < R_{crit}$ the diffusion of the H_2 adsorbed at the surrounding is the rate limiting step. Interestingly, the H_2 diffusion from the surrounding is always the rate limiting step at the end of the shrinking process when the islands are small enough.
However, the reduction process is only dominating for islands that are large enough. From (eq. 11) we see that if $\chi < 1$, then we have always $R_{crit} > R_0$ and therefore $R_{crit} > R$, meaning diffusion limitation. Only when $\chi > 1$ islands are large enough. From (eq. 7) we can calculate the minimum size the island must have initially, so in the beginning the reduction on the island surface is the rate limiting step:

$$R_0 > \frac{D_H}{k_{red}}$$

(37) Schmidt, T.; Sala, A.; Marchetto, H.; Umbach, E.; Freund, H. J. First experimental proof for aberration correction in XPEEM: Resolution, transmission enhancement, and limitation by space charge effects. *Ultramicroscopy* **2013**, *126*, 23-32. DOI: http://dx.doi.org/10.1016/j.ultramic.2012.11.004.
(38) Schmidt, T.; Marchetto, H.; Lévesque, P. L.; Groh, U.; Maier, F.; Preikszas, D.; Hartel, P.; Spehr, R.; Lilienkamp, G.; Engel, W.; et al. Double aberration correction in a low-energy electron microscope. *Ultramicroscopy* **2010**, *110* (11), 1358-1361. DOI: http://dx.doi.org/10.1016/j.ultramic.2010.07.007.
(39) Fink, R.; Weiss, M. R.; Umbach, E.; Preikszas, D.; Rose, H.; Spehr, R.; Hartel, P.; Engel, W.; Degenhardt, R.; Wichtendahl, R.; et al. SMART: a planned ultrahigh-resolution spectromicroscope for BESSY II. *Journal of Electron Spectroscopy and Related Phenomena* **1997**, *84* (1), 231-250. DOI: https://doi.org/10.1016/S0368-2048(97)00016-9.
(40) Mortensen, J. J.; Larsen, A. H.; Kuisma, M.; Ivanov, A. V.; Taghizadeh, A.; Peterson, A.; Haldar, A.; Dohn, A. O.; Schäfer, C.; Jónsson, E. Ö.; et al. GPAW: An open Python package for electronic structure calculations. *THE JOURNAL OF CHEMICAL PHYSICS* **2024**, *160* (9). DOI: 10.1063/5.0182685 (acccessed 2/10/2026).
(41) Hammer, B.; Hansen, L. B.; Nørskov, J. K. Improved adsorption energetics within density-functional theory using revised Perdew-Burke-Ernzerhof functionals. *Phys Rev B* **1999**, *59* (11), 7413-7421. DOI: 10.1103/PhysRevB.59.7413.
(42) Hjorth Larsen, A.; Jørgen Mortensen, J.; Blomqvist, J.; Castelli, I. E.; Christensen, R.; Dułak, M.; Friis, J.; Groves, M. N.; Hammer, B.; Hargus, C.; et al. The atomic simulation environment—a Python library for working with atoms. *Journal of Physics: Condensed Matter* **2017**, *29* (27), 273002. DOI: 10.1088/1361-648X/aa680e.
(43) Monkhorst, H. J.; Pack, J. D. Special points for Brillouin-zone integrations. *Phys. Rev. B: Solid State* **1976**, *13*, 5188.
(44) Apostol, T. M. *Calculus: One-Variable Calculus, with an Introduction to Linear Algebra*; John Wiley and Sons, Inc., 1967.
